# Electricity generation and real oily wastewater treatment by* Pseudomonas citronellolis* 620C in a microbial fuel cell: pyocyanin production as electron shuttle

**DOI:** 10.1007/s00449-024-03016-1

**Published:** 2024-04-17

**Authors:** Constantina K. Varnava, Panagiotis Persianis, Ioannis Ieropoulos, Argyro Tsipa

**Affiliations:** 1 Department of Civil and Environmental Engineering, University of Cyprus, Nicosia, Cyprus; 2 Water and Environmental Engineering Group, University of Southampton, Southampton, SO16 7QF UK; 3 Nireas International Water Research Centre, University of Cyprus, Nicosia, Cyprus

**Keywords:** Bioelectrochemical systems, Electricity generation, *P. citronellolis*, Pure cultures, Pyocyanin, Wastewater biodegradation

## Abstract

In the present study, the potential of *Pseudomonas citronellolis* 620C strain was evaluated, for the first time, to generate electricity in a standard, double chamber microbial fuel cell (MFC), with oily wastewater (OW) being the fuel at 43.625 mg/L initial chemical oxygen demand (COD). Both electrochemical and physicochemical results suggested that this *P. citronellolis* strain utilized efficiently the OW substrate and generated electricity in the MFC setup reaching 0.05 mW/m^2^ maximum power. COD removal was remarkable reaching 83.6 ± 0.1%, while qualitative and quantitative gas chromatography/mass spectrometry (GC/MS) analysis of the OW total petroleum and polycyclic aromatic hydrocarbons, and fatty acids revealed high degradation capacity. It was also determined that *P. citronellolis* 620C produced pyocyanin as electron shuttle in the anodic MFC chamber. To the authors’ best knowledge, this is the first study showing (phenazine-based) pyocyanin production from a species other than *P. aeruginosa* and, also, the first time that *P. citronellolis* 620C has been shown to produce electricity in a MFC. The production of pyocyanin, in combination with the formation of biofilm in the MFC anode, as observed with scanning electron microscopy (SEM) analysis, makes this *P. citronellolis* strain an attractive and promising candidate for wider MFC applications.

## Introduction

Over recent years, enhanced methods of detecting chemical contaminants have enabled measurement of pollutants concentrations in parts per billion in water, soil and sediment, as well as in the atmosphere. This capability has nevertheless revealed a host of challenges, such as the indiscriminate disposal and spreading of pollutants and the insufficient methods of waste storage, treatment, and disposal facilities, which contribute to contamination globally with severe environmental and human health consequences. Recognising the threat, governments are investing heavily to remediate increasing amounts of complex pollutants, as a consequence of economic development and rising living standards [1, 2]. Indicatively, the global environmental remediation market was valued at USD 110.68 billion in 2022, with the market being expected to reach USD 198.11 billion by 2030 [3]. Among the billions of pollutants, oily wastewater is considered a huge threat to the environment [4]. Components of the oily wastewater are oils, fats, greases, and multiple organic and/or inorganic dissolved substances. Such wastewater can be produced by a variety of industries such as petrochemical industries, edible oil refineries, drilling operation industries, metal processing industries, poultry processing industries, restaurants, dairy industries, slaughterhouses, tannery industries, etc. [5]. It is characterized by high concentrations of hydrocarbons such as total petroleum and polycyclic aromatic hydrocarbons, fatty acids and other toxic compounds.Therefore, oily wastewater treatment is essential.

Despite significant investments, the remediation of contaminated sites and water is still very challenging. Challenges are mainly due to the different-unique nature of the affected sites and the wide variety of pollutants [2, 6]; these can be inorganic or organic with some of the latter being highly toxic and extremely persistent [7], such as hydrocarbons and fatty acids. Furthermore, current applied technologies, apart from requiring large amounts of external energy and chemicals supply, are difficult and costly to manage [8, 9]. Compared with decontamination methods that either physically remove the pollutants or inactivate them by chemical detoxification or disinfection/sterilisation, biodegradation is considered cost-effective and more environmentally friendly. In particular, regarding oily wastewater treatment, among other treatments such as membrane separation technology, coagulation, flotation, combined technology and advanced oxidation process [4], biological treatment through biodegradation is considered a greener approach. However, the bioremediation of pollutants in the environment is, in most cases, relatively slow mainly due to the lack of suitable electron donors and/or acceptors, which are effective at oxidising contaminants [2, 6].

In the last 3 decades, microbial fuel cells (MFCs) have been extensively investigated as competitive alternatives to overcome the limitations of conventional remediation technologies [10, 11]. The huge advantage of MFCs compared with traditional physical, chemical, or even biological remediation methods lies in the use of electrodes as non-exhaustible electron acceptors/donors, for pollutant biodegradation. Therefore, the bioremediation time is significantly shorter, while very little or even no external energy and chemical supply is required. Instead, a good amount of energy is generated by the system itself [2, 6, 12, 13], which can serve as a real-time bioremediation indicator [14]. By shortening process times and reducing or even eliminating the need for external supply of energy and chemicals, the operational cost of the system is significantly reduced. In addition, MFCs provide both oxidation (anode) and reduction (cathode) reactions during which a multitude of microbial-electrochemical mechanisms is activated. As a result, MFCs have advantages in degrading complex pollutants with a variety of characteristics, in contrast to conventional remediation methods, which only provide one redox condition [13, 15, 16]. Oily wastewater has also been studied through electro-assisted biodegradation [17–19].

Despite the recognised high potential of MFCs in pollutant biodegradation, to date the technology is still limited to laboratory and pilot-scale applications. Scaling-up to industrial levels is still challenging mainly due to the lack of manufacturable materials and also knowledge on the gene regulation of electroactive microorganisms and their biodegradation capabilities in MFCs [6, 10, 11, 20]. To date, more than 100 different microorganisms, isolated from all three domains of life, can, with the right media and growth conditions, generate electricity in MFCs [21, 22]. The most well studied electroactive species are the gram-negative *Geobacter sulfurreducens* and *Shewanella oneidensis* [18]. They can produce high power densities due to their highly efficient extracellular electron transfer (EET) pathways. However, an extensive study of the EET and electro-assisted bioremediation molecular mechanisms by different electroactive microorganisms is crucial and will allow optimization of MFC technology. It has been proven that mixed cultures are highly efficient in generating electricity and adapting to the complex environment of MFC systems. However, it is the study of pure cultures that can elucidate the EET and electro-assisted biodegradation mechanisms in terms of the specific genetic and enzymatic steps followed. Therefore, these studies can reduce the complexity of understanding the microbial communities [23, 24] or assist when MFCs are used as biosensors for specific target analytes.

*Pseudomonas* is a metabolically versatile bacterium that can be found in diverse environments; hence, it is able to compete for a variety of substrates under both aerobic and anoxic conditions, and constitutes a particularly efficient anode biocatalyst in MFC systems with enormous potential for bioremediation of environmental pollutants [24–26]. *Pseudomonas* spp. are capable of degrading various substances of oily wastewater, such as aliphatic, mono-aromatic and polyaromatic hydrocarbons and fatty acids [27]. Such biodegradation metabolic pathways can occur simultaneously [28]. Under aerobic conditions, the metabolism of hydrocarbons is introduced using molecular oxygen as a co substrate in mono- or dioxygenase reactions which provide different types of hydroxylation of aliphatic chains and aromatic rings [27]. While under anaerobic conditions, the metabolism of hydrocarbons is introduced through fumarate addition and carboxylation of aliphatic chains and aromatic rings. The latter also go through hydroxylation [29]. Furthermore, fatty acids metabolism is introduced through the β-oxidation cycle under aerobic conditions [30]. Under anaerobic conditions, fatty acids are degraded using electron acceptors such as fumarate, nitrate or trimethylamine and β-oxidation cycle [31].

The most common *Pseudomonas* sp. studied in MFCs is *Pseudomonas aeruginosa* due to its diverse metabolic activity, application in waste treatment, and ability to produce compounds of significance such as antibiotics, siderophores, biosurfactants, etc*.* [25]. Other *Pseudomonas* spp. successfully applied in MFCs are *putida* [32, 33] and *fluorescens* [34, 35]. It was reported that phenazine-based metabolites such as pyocyanin and phenazine-1-carboxamide produced by some *Pseudomonas* spp., in most cases in a synergetic effect with biosurfactants. These molecules function as electron shuttles, enabling the bacterium to achieve its EET [36–39].

In this study, the electroactivity and biodegradation capability of *P. citronellolis* 620C; a strain recently isolated in our lab and shown to be highly capable of degrading oily wastewater [40, 41], was assessed. The electroactivity and biodegradation ability of this *P. citronellolis* strain was tested in medium-sized double-chamber MFC, fed with oily wastewater (OW) as a substrate. MFCs inoculated with *P. citronellolis* were considered as a unique opportunity to combine the metabolic capability of the strain to biodegrade OW [40, 41] with electricity generation. To the best of our knowledge, the MFC performance of *P. citronellolis* has not been previously studied. In addition to the simple calculation of the generated electrical energy, the biodegradation capability of *P. citronellolis* 620C in MFC conditions was evaluated through chemical oxygen demand (COD) removal calculation. Real OW; a highly toxic pollutant (COD ~ 43.625 mg/L), was characterized via GC/MS and the removal of its contained hydrocarbons (total petroleum hydrocarbons, TPHs and polycyclic aromatic hydrocarbons, PAHs) and fatty acids (FAs) was quantified. Finally, the presence of pyocyanin pigment was also established. The production of pyocyanin, in combination with biofilm formation that observed from scanning electron microscopy (SEM) analysis, explains the relatively high-power production from a pure bacterial culture using such a highly toxic wastewater.

## Materials and methods

### Microbial fuel cell construction and operation

A medium-sized 6 cm $\times$ 5 cm $\times$ 3 cm (h, w, d) double chamber microbial fuel cell (MFC), with a working volume of 25 mL for both the anode and cathode compartments (50 mL in total), was fabricated using polyacrylic material. The anode and cathode chambers were internally separated by a 6 $\times$ 5 cm cation exchange membrane (CEM, 125 $\times$ 125 mm, VWR Chemicals, UK). The anode and cathode electrodes used in this study were constructed from pieces of 9 cm $\times$ 30 cm plain carbon veil, folded down to 3 cm $\times$ 4 cm to fit inside the MFC framework. The anolyte was filled with 25 mL medium and pure culture of *P. citronellolis* 620C. The catholyte was potassium ferricyanide, [K_3_Fe(CN)_6_], 0.02 M, and from the 15th operation day onwards 0.5 M in potassium phosphate buffer, pH 7. The MFC was operated in both open circuit voltage (OCV) and closed circuit voltage (CCV) operating conditions; for the latter, the anode and cathode electrodes were connected with a resistor, whose value was determined depending on experiment and from polarisation. The initial resistor value was 4.5 k*Ω*. Both the anode and cathode were set at a pH of 7. The MFC was operated in fed-batch mode, whereby the feeding was carried out when the output voltage decreased below baseline, under sterile semi-anaerobic conditions. On the other hand, the catholyte from the 15th operation day onwards was regularly changed every 7 days upon intense darkening observation. Following polarization study, the resistor value was set to 20 k*Ω*. Subsequently, on the 55th day, after the system demonstrated stable performance for an extended duration, the resistance was significantly reduced from 20 to 9 k*Ω*, followed by conservative adjustments at fixed time intervals until reaching the initial applied resistance of 4.5 k*Ω*. All MFC components were sterilised before use. No external electron mediators were used during the study.

### Bacterial strain and growth conditions

The inoculum consisted of a pure culture of the *P. citronellolis* 620C, with the accession number PP237391 in genbank. *P. citronellolis* 620C was retrieved from a glycerol stock (stored microorganism in 25% (*v/v*) glycerol) preserved at − 80 °C, and then cultivated within the MFC anodic chamber at 30 °C and 100 rpm shaking conditions (Orbital Shaker Incubator, MRC ltd.). The growth medium comprised of 10% (*v*/*v*) M9 minimal medium prepared with 33.91 g Na_2_HPO_4_, 15 g KH_2_PO_4_, 15 g NaCl, 5 g NH_4_Cl in 500 mL dH_2_O, supplemented with and 0.02% (*v*/*v*) MgSO_4_ (12.0372 g in 100 mL dH_2_O), along with 1% (*v*/*v*) oily wastewater (OW, IESC company, Limassol, Cyprus). The OW came from the drilling operations at the Cypriot sea [40]. The 1% (*v*/*v*) OW was introduced to the anode in the beginning of the experiment and when deemed necessary based on the output voltage during the period of 70 days of operation.

### Measurement and analysis

#### Electrical measurements

The MFC was, initially, allowed to reach a stable OCV for approximately 3 h, before applying a 4.5 k*Ω* resistor as external load. The electrical output was manually recorded as volts (*V*) against time, using a standard digital multimeter [Topex, Universal Electronic Multimeter, 70-94W105]. The current (*I*), in amperes (*A*), was calculated according to Ohm’s law:

1 $$I=V/R$$

where, *V* is the measured voltage in volts (*V*) and *R* is the known value of the external load resistor in Ohms (*Ω*).

The power (*P*), in watts (*W*) was calculated as the product of the measured voltage and calculated current as follows:

2 $$P=I \times V$$

Polarisation experiments were carried out using a variable resistance box in a laminar flow cabinet at room temperature. Prior to these experiments, the MFC was allowed to reach a stable OCV. Polarisation data were produced by varying external resistances from 90 k*Ω* to 100 *Ω* and the time interval between resistance changes was 5 min.

#### Physicochemical analysis

##### Chemical oxygen demand (COD)

Chemical oxygen demand was measured using a Supelco Inc Sigma-Aldrich, St. Louis, MI, USA kit with a range between 500 and 10.000 mg/L. Briefly, 1 mL of the $\times$ 5 diluted anolyte sample was added to the vial, which was vigorously mixed and then heated at 148 °C in a pre-heated thermoreactor (CR 3200, WTW, Xylem Analytics, Weilheim, Germany) for 120 min. Following cooling down at room temperature, the COD concentration was measured in a spectrophotometer (Spectroquant^®^ Prove Spectrophotometer 100, Merck Millipore, Darmstadt, Germany). Considering the 5 × dilution, the obtained COD value was, then, multiplied by 5. The control for the COD measurement contained the medium and 1% (*v/v*) OW.

##### Scanning electron microscopy (SEM)

At the end of the experiment, scanning electron microscopy was destructively performed. The anodic chamber was opened, and the electrode was fixed in 4% glutaraldehyde in sterile phosphate buffer saline (PBS) for 1 h at room temperature. The electrode was then rinsed in PBS for three 1 h periods before storing at 4 °C overnight. Then, the electrode was dehydrated by a graded ethanol series (30, 50, 70, 80, 95, and 100%; at 5 min stages), and then vacuum dried. Representative samples from the electrode were prepared for microscopy by mounting on double-sided carbon tape and sputter-coating in gold [20]. Images were recorded using a JEOL, JSM-6610 LV scanning electron microscope (SEM) equipped with a BRUKER type QUANTAX 200 energy dispersive X-ray spectrometer (EDS). The control for the SEM analysis was a blank electrode.

##### Gas chromatography/mass spectrometry (GC/MS)

*i. Total petroleum hydrocarbons, TPHs*

A total of 1 mL of the anolyte was transferred into a test tube and extracted with 5 mL *n*-hexane. Following extraction, the organic (upper) phase was collected and a small amount of desiccant, anhydrous MgSO_4_, was added to ensure dehydration. Prior to GC/MS analysis, the final solution was filtered and transferred into a 2 mL GC vial.

The TPHs were analyzed by a 8060 GC/5977B MSD (Agilent Technologies, Inc., Santa Clara, CA, USA) equipped with an Agilent HP-5MS Ultra Inert capillary column (30 m long × 0.25 mm ID × 0.25 μm film thickness). The temperature of the ion source was set at 230 °C with 70 eV of ionization energy. Helium (99.999%) was used as carrier gas at a flow rate of 1.0 mL/min. The injector temperature was maintained at 250 °C with the following ramp-up conditions: 50 °C kept constant for 1 min; increased up to 110 °C with 10 °C/min rate and 1 min hold; further increased up to 270 °C with 3 °C/min rate and 1 min hold; followed by a final increase at 300 °C with 15 °C/min rate and 10 min hold (74 min total run time). The injection was splitless with a volume of 1.0 μL. MS was performed at scan mode over the 46–800 m*/z* range. The MS spectra obtained for the TPHs was matched to the US National Institute of Standards and Technology (NIST) [42] mass spectral library database.

*ii. Polycyclic aromatic hydrocarbons, PAHs*

The extraction of polycyclic aromatic hydrocarbons (PAHs) was carried out with the method mentioned in TPHs analysis section. The extracted sample was analyzed on the 8060 GC/5977B MSD equipped with the Agilent HP-5MS Ultra Inert capillary column. The temperature of EI ion source was set at 230 °C with 70 °eV of ionization energy. Helium (99.999%) was used as carrier gas at a flow rate of 1.0 mL/min. The injector temperature was maintained at 320 °C with the following oven conditions: 80 °C kept constant for 1 min; increased up to 200 °C with 25 °C/min rate; further increased up to 335 °C with 8 °C/min rate and 6.325 min hold (29 min total run time). The injection was splitless with a volume of 1.0 μL. MS was performed at scan and SIM positive ion mode over the 75–300 m*/z* range. The MS spectra obtained for the PAHs was matched to the US NIST [42] mass spectral library database.

*iii. Fatty acids methyl esters, FAMEs*

A total of 100 μL anolyte sample was methylated and converted to FAMEs following the procedure below [43]: One milliliter of *n*-hexane was added to 100 μL of anolyte sample. Thereafter, 1 mL of sodium methoxide 1 M (2 g of NaOH in 50 mL methanol) solution was added and the mixture was vortexed for 30 s. The solution was, then, centrifuged at 1200 rpm and incubated at room temperature for 10 min to separate out the clear layer solution containing FAMEs from the turbid aqueous layer. Finally, a small amount of a desiccant, anhydrous MgSO_4_, was added to the obtained sample to ensure its dehydration. Prior to GC/MS analysis, the final solution was filtered and then transferred into a 2 mL GC vial.

The FAMEs were analyzed by a 8060 GC/5977B MSD equipped with an Agilent HP-5MS Ultra Inert capillary column. The temperature of EI ion source was set at 230 °C with 70 eV of ionization energy. Helium (99.999%) was used as carrier gas at a flow rate of 1.0 mL/min. The injector temperature was maintained at 250 °C with the following oven conditions: 50 °C kept constant for 1 min; increased up to 200 °C with 10 °C/min rate and 1 min hold; further increased up to 230 °C with 3 °C/min rate; followed by 23 min hold at 230 °C (50 min total run time). The injection was splitless with a volume of 1.0 μL. MS was performed at scan and SIM positive ion mode over the 46–500 m*/z* range [44]. The MS spectra obtained for the FAMEs was firstly matched to the US NIST [42] mass spectral library database and, then identified and quantified using established calibration curves.

### Extraction of pyocyanin from *P. citronellolis* 620C

A loop-full sample of the blue-greenish anolyte was aseptically transferred from the anolyte to 50 mL lysogeny broth (LB) in a 250 mL conical flask and incubated with shaking at 30 °C for 24 h. The 24-h culture was then centrifuged at 10,000 rpm for 20 min at 4 °C and the supernatant was collected and transferred into a 500 mL separating funnel and mixed with chloroform at a 1:2 ratio (supernatant: chloroform) forming two-separating phases. The blue-coloured chloroform layer contained the formed pyocyanin, below the aqueous layer. The blue-coloured phase was collected into a covered conical flask to protect from light to prevent oxidation and concentrated with 0.1 M HCl under continuous stirring until the whole blue pigment was converted into the acidic form (red). Then, the acidified layer pH was neutralised to level 7 by using 1 M NaOH [45, 46].

### Characterisation of pyocyanin from *P. citronellolis* 620C under MFC operation conditions

#### Ultraviolet–visible (UV–Vis) spectroscopy

The extracted pyocyanin was subjected to UV–Vis spectroscopic analysis. UV–Vis absorption spectra of the acidified and neutral extracted pyocyanin were recorded over a range of 200–800 nm [47, 48]. UV–Vis analysis was done using UV–Visible spectrophotometer (JASCO V-530 PC, Nicosia, Cyprus).

#### Gas chromatography/mass spectrometry (GC/MS)

GC/MS analysis of the extracted pyocyanin was performed by a 8060 GC/5977B MSD, under these conditions [47]: Agilent HP-5MS Ultra Inert capillary column; oven temperature program: the column held initially at 50 °C and kept constant for 2 min; increased to 150 °C with a 7 °C/min heating ramp; further increase up to 270 °C with 5 °C/min rate and 2 min hold; followed by a final increase to 310 °C with a 7 °C/min heating ramp and 10 min hold (64 min total run time). The injection was operated in splitless mode. The temperature of EI ion source was set at 230 °C with 70 eV of ionization energy. Helium (99.999%) was used as carrier gas at a flow rate of 1.0 mL/min. The injector temperature was maintained at 280 °C. The injection was splitless with a volume of 1.0 μL. MS was performed at scan and SIM positive ion mode over the 34–450 m*/z* range [44]. The MS spectra obtained was matched to the US NIST [42] mass spectral library database.

## Results and discussion

### Polarisation study

**Fig. 1:**
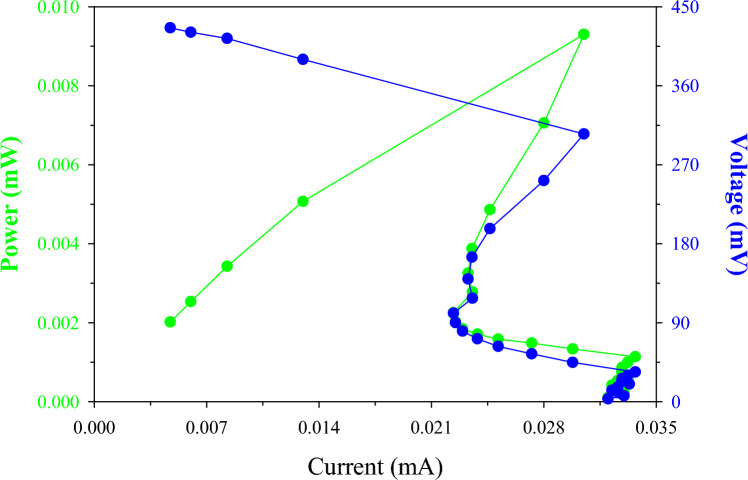
Power and polarisation data for the double chamber MFC operated with oily wastewater

After establishing pseudo steady-state in open circuit voltage (OCV) conditions, the effect of the external resistance on MFC operation was examined by varying the resistive load from 90 k*Ω* to 100 *Ω*. The OCV from the MFC was 432 mV. Output voltage data were recorded for each resistor value every 5 min (see Fig. 1). As can be seen, a maximum power of 9 μW (peak *P*) from the MFC, with a corresponding current of 31 μ*A*, was obtained at 20 k*Ω* followed by a sharp decrease of power output. Similar polarization studies have observed such behaviour of the system [49]. Maximum power and current was produced at 305 mV; this was higher than ½ OCV, which is the operating level for maximum power transfer. It is quite clear that the maximum value was the result of power overshoot, a phenomenon that has been previously reported to be a function of system immaturity, starvation, ion depletion and internal resistance [50]. In this case, the system was arguably well established, so one explanation for this overshoot behaviour could be ion depletion, due to the use of OW or culture starvation due to the irregular feeding intervals.

### Energy generation—performance of the MFC

**Fig. 2:**
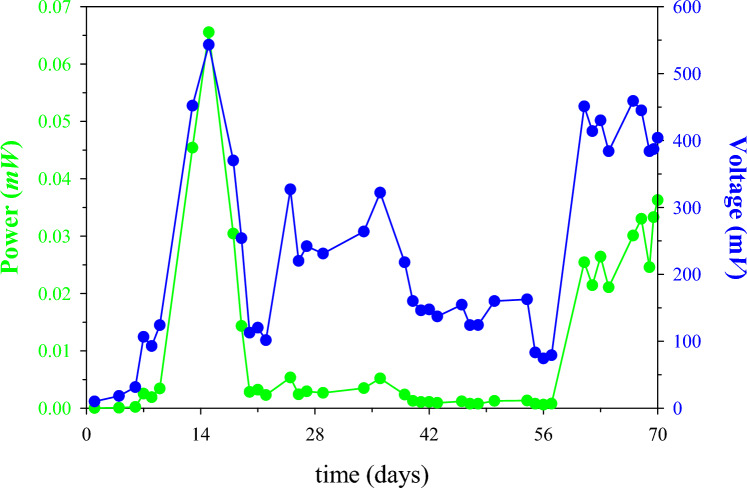
Power and voltage of the double chamber MFC. Catholyte concentration was increased on day 15 and changed every 7 days, and polarisation was carried out on day 22

**Fig. 3:**
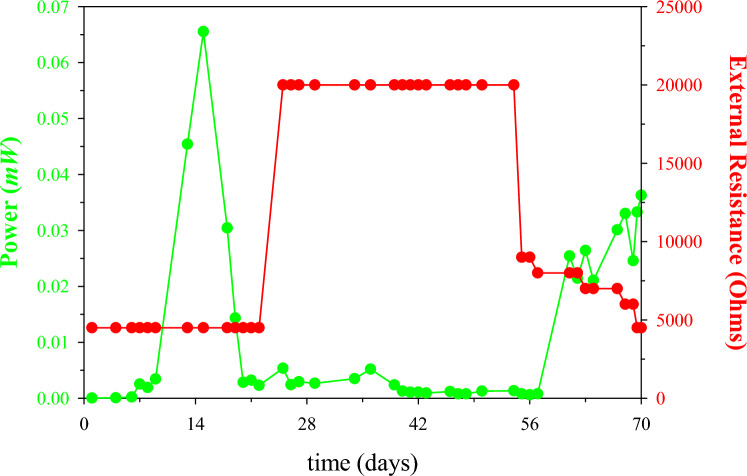
Temporal power data**.** Catholyte concentration was increased on day 15 and changed every 7 days, and polarisation was carried out on day 22

Temporal data over the full 70-days duration of the experiment are shown in Fig. 2. On day 15 the concentration of the K_3_Fe(CN)_6_ buffer solution in the cathode was increased from 0.02 to 0.5 M, to be consistent with the majority of the reported studies in the literature [51–53]. Subsequently after that change, the highest CCV was observed at 543 mV (1.8 W/m^3^ or 0.05 mW/m^2^). Furthermore, following polarisation study carried out on day 22, a 200% increase in output voltage data (from 101.5 to 327 mV) was observed (Fig. 3).

Figure 3 shows the amount of power produced during the sequential decreases of the external resistance. Following the changes in catholyte concentration and the polarisation, an almost stable MFC performance, in terms of CCV and electricity generation, was observed by applying the optimum load resistor (Fig. 3). Subsequently, on the 55th day of the MFC operation, the response of the MFC was evaluated by decreasing the external resistance load at regular time intervals.

A 20-fold increase in electricity generation was observed when the resistance was decreased from 20 to 8 k*Ω*. When the load was further reduced to 4.5 k*Ω*, power output increased 30x. The higher power production can perhaps be attributed to healthy biofilm formation over time on the anode electrode [54–56]. Following this simple optimisation, the power output increase on day 70 was 3 orders of magnitude higher, under the same 4.5 k*Ω* load that was applied in the beginning of the experiment (day 1).

**Fig. 4:**
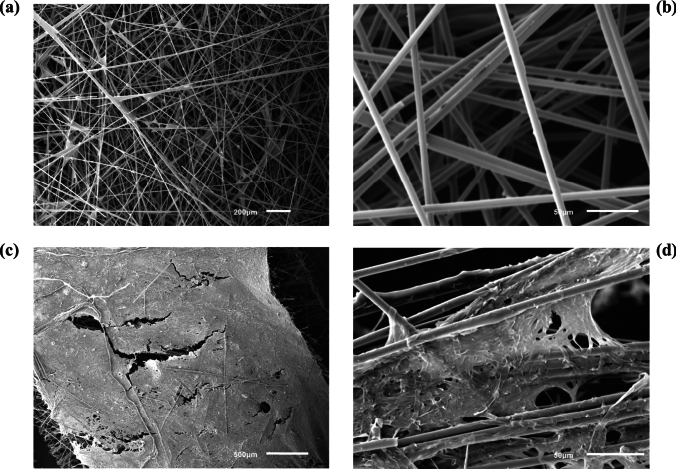
SEM images **a** 200 μm and **b** 50 μm of a blank electrode, **c** 500 μm and **d** 50 μm of the anode electrode surface after 70 days of MFC operation with OW

SEM analysis on the electrode surface before (Fig. 4a, b) and after the experiment (Fig. 4c, d) showed successful biofilm formation, which can possibly explain the improvement in power output [20, 57]. A firm film is observed at lower magnification (500 μm) (Fig. 4c) and a more detailed structure of the biofilm is observed at 10 × fold magnification (50 μm) (Fig. 4d), when compared to similar magnification of the electrode surface before the experiment. Although higher magnification (which would have revealed individual cells) was not possible on this occasion, the images after 70 days of operation are consistent with previously reported images showing biofilm formation of *Pseudomonas* genus species [58].

The COD reduction was calculated during MFC operation with 4.5 k*Ω* resistance on day 70 of operation and 7 days after the last feed replacement of the anode with OW. COD reduction efficiency was 83.6 ± 0.1% and calculated against the control sample at an almost stabilized CCV of 404 mV (40 μW power/1 W/m^3^ or 0.03 W/m^2^). This high COD removal is attributed to the homogeneous biofilm formation observed in the anodic compartment (SEM analysis), which also contributed to the high-power outputs. COD removal using different types of OW biological treatment ranges from 82 to 97% [4]. Interestingly, in such biological treatments, mixed cultures are used, which it is recognized, that can achieve higher biodegradation efficiencies compared to pure cultures. Similarly, upon OW biodegradation using MFC technology and mixed cultures, the COD removal efficiency was within 40 to 87% [17, 19]. In the current study, where a pure culture of *P. citronellolis* was used, COD removal was within the same range. Furthermore, it is noteworthy that another study of OW treatment in MFC using *P. putida* achieved just 30% COD removal with initial COD of 2213 mg/L in an air–cathode MFC [32]. The present *P. citronellolis* strain achieved almost 84% COD removal with an initial COD of 43.625 mg/L. The typical dual chamber MFC which has some practical deficiencies compared to air–cathode MFCs was used. Therefore, the OW removal efficiency of the current strain is remarkable. In addition, bio-electrochemical degradation presents significant industrial advantages thanks to faster rates of degradation, compared to traditional biodegradation, as well as concurrent electricity generation. By harnessing waste as a substrate in bioelectrochemical systems, it serves as a “catalyst” for electricity production, aligning with the principles of the circular economy and green chemistry, wherein wastewater is transformed into a valuable resource. Furthermore, such systems are greener, more environmentally friendly, energy- and cost-effective when compared to conventional oily wastewater treatment processes.

### Biodegradation of hydrocarbons and fatty acids

#### i. Total petroleum hydrocarbons, TPHs and polycyclic aromatic hydrocarbons (PAHs)

**Fig. 5:**
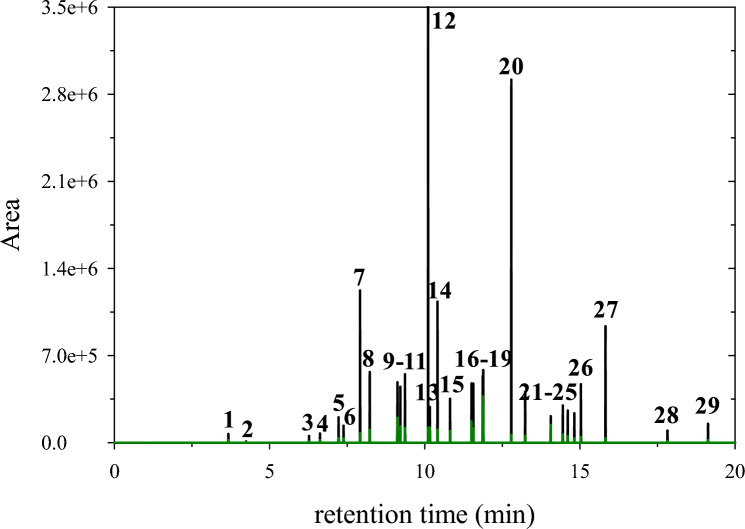
Total Petroleum Hydrocarbons (TPHs) total ion chromatogram where 29 TPHs were detected in OW **(black)** and MFC anolyte before the feed replacement with OW **(green)**. Peaks 1–29 correspond to TPHs listed in Table 1

TPHs were calculated on the 70th day of operation and 7 days after the last feed replacement of the anode with OW. GC coupled with MS was used to identify the TPHs contained in OW. The analysis revealed that OW consisted mainly of *n*-alkanes C9 to C15, with intermediate branched chain hydrocarbons, and other petroleum-based compounds, cyclic and aromatic. A set of 29 peaks is discernible in the total ion chromatogram illustrated in Fig. 5 and listed in Table 1.

**Table 1:** Quantification results of total petroleum and polycyclic aromatic hydrocarbons contained in the OW of MFC anolyte before and after feed

# Peak	TPH	Molecular formula	Retention time (min)	Area		Removal rate (%) ^a^
				In OW	In MFC anolyte	
1	2,4-dimethyl heptane	C_9_H_20_	3.68	69,798	20,854	70.1
2	4-methyl octane	C_9_H_20_	4.24	10,720	8596	19.8
3	*n*-decane	C_10_H_22_	6.27	53,329	10,389	80.5
4	4-methyl decane	C_11_H_24_	6.62	73,649	19,771	73.2
5	*trans*-decalin	C_10_H_18_	7.23	203,256	35,792	82.4
6	3-methyl decane	C_11_H_24_	7.39	136,110	32,788	75.9
7	*n*-undecane	C_11_H_24_	7.92	1,222,849	74,816	93.9
8	*trans*-2-methyl decalin	C_11_H_20_	8.23	567,192	102,439	81.9
9	4-methyl undecane	C_12_H_26_	9.12	484,740	199,418	58.9
10	2-methyl undecane	C_12_H_26_	9.22	448,613	130,450	70.9
11	3-methyl undecane	C_12_H_26_	9.37	549,365	118,277	78.5
12	*n*-dodecane	C_12_H_26_	10.11	3,499,469	120,193	96.6
13	2,6-dimethyl decalin	C_12_H_22_	10.17	286,648	121,924	57.5
14	2,6-dimethyl undecane	C_13_H_28_	10.41	1,133,619	104,463	90.8
15	*trans*,*cis*-2-ethylbicyclo[4.4.0]decane	C_12_H_22_	10.82	352,891	92,929	73.7
16	1,3-bis(1,1-dimethylethyl)benzene	C_14_H_22_	11.51	475,408	171,886	63.8
17	4-methyl dodecane	C_13_H_28_	11.57	474,836	114,559	75.9
18	3-methyl dodecane	C_13_H_28_	11.87	534,342	371,708	30.4
19	2,9-dimethyl undecane	C_13_H_28_	11.89	583,725	289,879	50.3
20	*n*-tridecane	C_13_H_28_	12.79	2,917,043	61,576	97.2
21	6-methyl tridecane	C_14_H_30_	13.24	408,199	53,433	84.9
22	cyclotridecane	C_13_H_26_	14.06	212,945	142,855	74.9
23	4-methyl tridecane	C_14_H_30_	14.45	298,094	66,327	52.1
24	2-methyl tridecane	C_14_H_30_	14.61	257,815	50,228	74.3
25	3-methyl tridecane	C_14_H_30_	14.82	235,882	34,619	78.7
26	2,6,11-trimethyl dodecane	C_15_H_32_	15.03	469,241	46,313	92.6
27	*n*-tetradecane	C_14_H_30_	15.83	935,423	35,591	95.1
28	2,6,10-trimethyl dodecane	C_15_H_32_	17.82	96,627	13,717	63.2
29	*n*-pentadecane	C_15_H_32_	19.13	151,902	20,854	91.0
30	naphthalene	C_10_H_8_	4.47	2,995,734	nd^b^	100
Mean removal rate (%)						74.3

^**a**^According to GC chromatogram areas

^**b**^Not detected

*P. citronellolis* 620C appears to have degraded TPHs, under MFC operating conditions. Several TPHs were almost undetected by GC/MS (Fig. 5—**green**). Table 1 presents the quantification results of GC/MS analysis.

PAHs were also calculated on the 70th day of operation and 7 days after the last feed replacement of the anode with OW. GC/MS analysis exclusively detected naphthalene in OW (retention time = 4.47 min, data not shown), with this PAH being completely decomposed during OW biodegradation and therefore not detected in the sample of MFC anolyte (Table 1).

The mean hydrocarbons removal rate was 74.3%, with some of them, especially the *n*-alkanes, reaching removal rates > 90%. Both aromatic and aliphatic hydrocarbons were biodegraded. The present *P. citronellolis* strain was operated under semi-anaerobic conditions. Therefore, further research is essential to elucidate the metabolic pathways followed for the biodegradation of such compounds in those conditions.

#### ii. Fatty acids methyl esters (FAMEs)

**Fig. 6:**
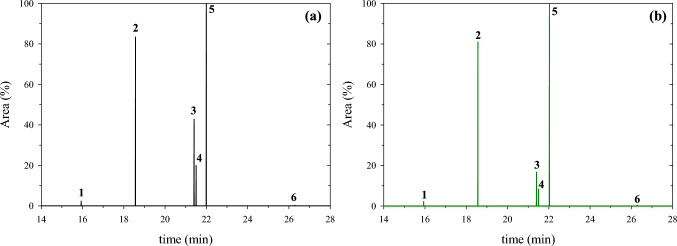
Fatty Acid Methyl Esters (FAMEs) analysis; Ion chromatograms at *m/z* = 74 where six Fatty Acids were detected in **a** OW **(black)** and **b** in MFC anodic chamber **(green)**. Peaks 1–6 correspond to methyl myristate (C14:0), methyl palmitate (C16:0), methyl linoleate (C18:2n6c), methyl elaidate (C18:1n9t), methyl stearate (C18:0), and methyl arachidate (C20:0), respectively

Fatty acids (FAs) were calculated on the 70th day of operation and 7 days after the last feed replacement of the anode with OW. FAs were subjected to methyl-esterification and the obtained FAMEs were analyzed by GC/MS. The removal rates of these FAs at high levels of power generation were also quantified. Figure 6 shows the extracted FAME ion chromatogram of *m/z* = 74; the characteristic peak for FAMEs due to the McLafferty rearrangement [59, 60], and reveals that the OW (Fig. 6a) consists of both saturated and unsaturated fatty acids. These are the methyl myristate (C14:0), methyl palmitate (C16:0), methyl linoleate (C18:2n6c), methyl elaidate (C18:1n9t), methyl stearate (C18:0), and methyl arachidate (C20:0). These results are consistent with levels reported in the literature [43, 61] regarding oily substrates. Quantitative analysis (Table 2) revealed that the major FAMEs in OW are palmitic (17.65% of total FAMEs concentration), linoleic (31.47% of total FAMEs concentration), elaidic (13.47% of total FAMEs concentration), and stearic (36.39% of total FAMEs concentration) acids, whereas methyl myristate and methyl arachidate were identified in significantly lower concentrations.

**Table 2:** Quantification results of FAMEs contained in oily wastewater and in MFC anolyte before feed replacement with oily wastewater

# Peak	FAME	Lipid number	Retention time (min)	Concentration (ppm)		Removal rate (%)
				In OW	In MFC anolyte	
1	Methyl myristate	C14:0	15.94	7.09	11.69	
2	Methyl palmitate	C16:0	18.55	252.49	414.06	
3	Methyl linoleate	(C18:2n6c)	21.41	450.03	300.50	33.23
4	Methyl elaidate	(C18:1n9t)	21.50	192.88	138.54	28.17
5	Methyl stearate	C18:0	22.00	520.40	880.74	
6	Methyl arachidate	C20:0	26.06	7.30	10.25	
Total FAMEs concentration				1430.20	1755.78	

According to calibration results

*P. citronellolis* 620C appears to have effectively diminished the unsaturated FAs of methyl linoleate and methyl elaidate (Fig. 2b and Table 2). However, the concentrations of saturated ones to anolyte sample were significantly higher (Table 2). This result can perhaps be attributed to the formation of intra and extra-cellular metabolites in the anodic compartment over time such as polyhydroxyalkanoates (PHAs) and biosurfactants, respectively. The biodegradation of the unsaturated FAs drive further research over the metabolic pathway followed in the semi-anaerobic conditions of the MFC operation.

### Characterisation of pyocyanin from *P. citronellolis* 620C

After approximately 35 days of MFC operation, it was observed that the turbid solution in the anodic compartment changed to blue-green. Therefore, it was considered beneficial to investigate the presence of phenazine pigment pyocyanin.

**Fig. 7:**
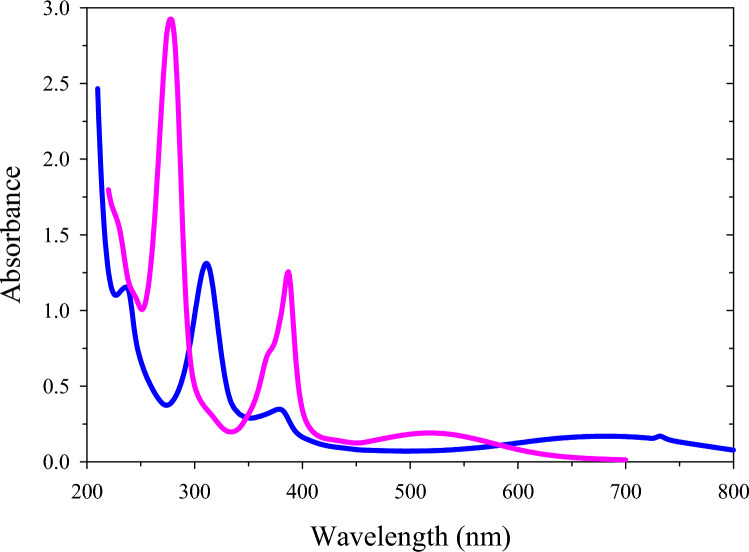
UV–Vis absorption spectra of acidified (pink) and neutral (blue) pyocyanin pigment isolated from *P. citronellolis* 620C under MFC operating conditions

The absorbance spectrum of pyocyanin was monitored from 200 to 800 nm using a UV–Vis spectrophotometer (Fig. 7) to determine the wavelength absorption of pyocyanin pigment produced by *P. citronellolis* 620C. UV–visible spectroscopic analysis of acidified (pink spectrum) pyocyanin showed three maxima at wavelengths 518, 387 and 278 nm. The UV–Vis spectrum of the neutral pyocyanin (blue spectrum) showed five maxima at wavelengths 732, 683, 378, 311 and 236 nm. The maximum peaks at 278 and 311 nm indicated the presence of the pyocyanin compound in the acidified and neutral sample, respectively.

These results are consistent with those from previously published studies. Koyun et al. [47] reported the presence of pyocyanin pigment in pure water at 300 nm. Sudhakar et al*.* [62] indicated that the maximum absorbance of the pyocyanin acidified solution was at 278 nm, while Priyala [63] found that UV–Vis spectroscopic analysis of pyocyanin observed four absorption maxima in 0.1 M HCl at wavelengths of 553 nm, 390 nm, 284 nm, and 246 nm.

**Fig. 8:**
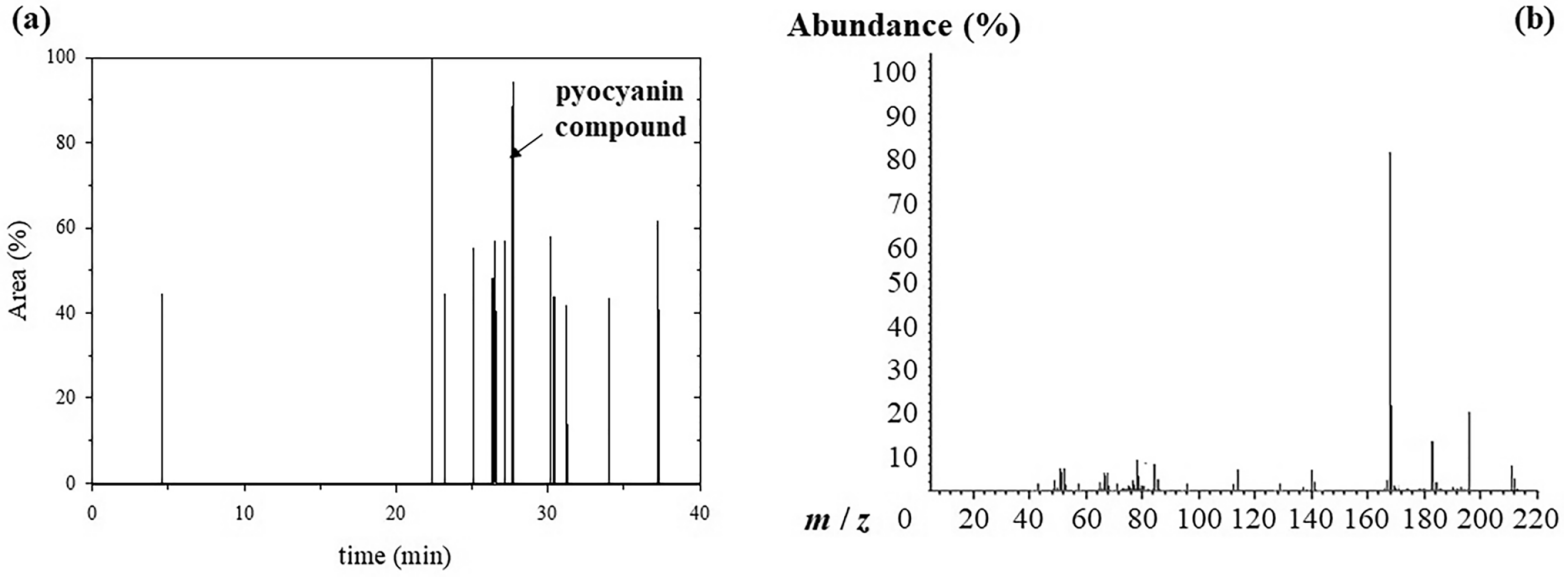
GC/MS analysis of pyocyanin pigment isolated from *P. citronellolis* 620C under MFC operating conditions **a** GC chromatogram and **b** MS spectrum

Figure 8 presents the GC/MS analysis of pyocyanin pigment produced by *P. citronellolis* 620C. The pyocyanin compound was detected at a retention time of 27.71 min (Fig. 8a), while its presence in the sample was confirmed by the dense molecular ion peaks at 168 and 196 of its *m/z* fractions in the MS spectrum (Fig. 8b). The sharp peak at 168 m*/z* is attributed to phenazine, while 1-hydroxyphenazine (hemi pyocyanin) exhibits a sharp peak at 196 m*/z*. However, the simultaneous detection of both molecular ion peaks in the MS spectrum demonstrates the presence of 1-hydroxy-*N*-methyl phenazine, “pyocyanin pigment”, which is converted to 1-hydroxyphenazine ([M-15]^+^ represents the loss of a methyl radical (•CH_3_)) [47].

Pyocyanin is an extracellular phenazine pigment produced by *P. aeruginosa* as a secondary metabolite and has a variety of biological actions. Indicatively, pyocyanin is characterised by antimicrobial, anticancer, antioxidant, antimalarial, antiparasitic and immunosuppressive properties. Due to these features, pyocyanin can be exploited for medical, pharmaceutical, food, textile, biocontrol, nanotechnology, and physicochemical applications [35, 36, 51, 53]. Pyocyanin can also be used as electron shuttle in MFCs enabling bacterial electron transfer towards the anode [45, 65]. This ability to support electron transfer makes pyocyanin an attractive candidate for use in various biotechnological applications beyond MFCs, such as biosensors in nanotechnology, colorimetric redox indicators, luminescence-based pH sensors, and organic light emitting devices [46, 64].

Synthetic dyes are widely utilised in many industrial sectors such as textile, paper, cosmetics, food, plastic, pharmaceutical products, etc*.*, with their global production being nearly 8 $\times$ 10^5^ tons per year. It is estimated that 10–15% of this production is lost in wastewater during manufacturing and application processes, making dyes the most discharged pollutants, amongst various industrial wastes [46, 66, 67]. The contamination of dyes in wastewater poses a threat to environment and public health due to their non-biodegradable complex molecular structures and mutagenic and carcinogenic, and allergenic to human body characteristics. Given the absence of a remediation technology that completely removes such persistent pollutants, natural pigments are a very attractive alternative. Natural pigments can be obtained from plants, microorganisms, and natural mineral sources [68]. However, pigments produced by microorganisms are preferred because of their photo-, thermal- and pH-stability and bio-availability [69].

As the produced pyocyanin pigment comprises of methyl palmitate (*t*_R_ = 26.54 min) and methyl stearate (*t*_R_ = 30.22 min), its application spectrum could further expanded. In addition to exceptional properties of pyocyanin compound, the simultaneous presence of methyl palmitate adds anti-inflammatory and antifibrotic features, permitting the exploitation from *P. citronellolis* 620C produced pyocyanin for medical and pharmaceutical applications [70]. The presence of fatty acid methyl esters, in general, makes pyocyanin a valuable candidate for use in cosmetics and cleaning products as colour additive [45].

The production of pyocyanin as electron shuttle, from *P. citronellolis* 620C under MFC conditions has been reported for the first time and it appears to be the result of (or indeed the reason why) higher power output was observed over time. Further work is clearly required to study the effects of pH, medium type and concentration, continuous flow, temperature, light or dark condition, etc. [45].

## Conclusions

This study showed for the first time that *P. citronellolis* 620C is electrochemically active inside MFCs, producing electricity and pyocyanin, over a period of 70 days; for a monoculture supported by biofilm formation, this is comparatively an acceptable level of performance, especially considering the high COD reduction efficiency and removal of OW compounds of TPHs, PAHs and FAs. These results show yet another attribute of the MFC technology, opening the opportunity for wider implementation in otherwise difficult environments. The MFC technology contributes directly towards the achievement of global strategic objectives, namely sustainability, resilience, net zero energy generation and environmentally-friendly waste treatment and depollution. Further optimisation studies need to be carried out, starting with better understanding of the bacterial metabolism. The choice of a pure culture in the MFC system facilitates this task, which is considered crucial for the employment of optimisation and engineering strategies.

## Data Availability

Data will be made available on request.

## Abbreviations

MFC: Microbial Fuel Cell
EET: Extracellular Electron Transfer
OW: Oily Wastewater
COD: Chemical Oxygen Demand
TPHs: Total Petroleum Hydrocarbons
PAHs: Polycyclic Aromatic Hydrocarbons
FAs: Fatty Acids
CEM: Cation Exchange Membrane
CCV: Closed Circuit Voltage
OCV: Open Circuit Voltage
FAMEs: Fatty Acid Methyl Esters
SEM: Scanning electron microscopy
GC/MS: Gas Chromatography/Mass Spectrometry

## References

[1] Khan S, Naushad M, Govarthanan M, Iqbal J, Alfadul SM (2022). Emerging contaminants of high concern for the environment: current trends and future research. Environ Res.

[2] Wang H, Luo H, Fallgren PH, Jin S, Ren ZJ (2015). Bioelectrochemical system platform for sustainable environmental remediation and energy generation. Biotechnol Adv.

[3] 2023 Environmental Remediation Market Size & Forecast Report 2030 *P & S Intelligence Market Research* https://www.psmarketresearch.com/market-analysis/environmental-remediation-market# (accessed Aug. 22, 2023).

[4] Yu L, Han M, He F (2017). A review of treating oily wastewater. Arab J Chem.

[5] Adetunji AI, Olaniran AO (2021). Treatment of industrial oily wastewater by advanced technologies: a review. Appl Water Sci.

[6] Wang X (2020). Microbial electrochemistry for bioremediation. Environ Sci Ecotechnology.

[7] Kucharzyk KH, Darlington R, Benotti M, Deeb R, Hawley E (2017). Novel treatment technologies for PFAS compounds: a critical review. J Environ Manage.

[8] Sevda S, Sreekishnan TR, Pous N, Puig S, Pant D (2018). Bioelectroremediation of perchlorate and nitrate contaminated water: a review. Bioresour Technol.

[9] Zou Y (2016). Environmental remediation and application of nanoscale zero-valent iron and its composites for the removal of heavy metal ions: a review. Environ Sci Technol.

[10] Santoro C, Arbizzani C, Erable B, Ieropoulos I (2017). Microbial fuel cells: From fundamentals to applications. a review. J Power Sources.

[11] Tsipa A, Varnava CK, Grenni P, Ferrara V, Pietrelli A (2021). Bio-electrochemical system depollution capabilities and monitoring applications: models, applicability, advanced bio-based concept for predicting pollutant degradation and microbial growth kinetics via gene regulation modelling. Processes.

[12] Xing W, Li D, Li J, Hu Q, Deng S (2016). Nitrate removal and microbial analysis by combined micro-electrolysis and autotrophic denitrification. Bioresour Technol.

[13] Huang DY, Zhou SG, Chen Q, Zhao B, Yuan Y, Zhuang L (2011). Enhanced anaerobic degradation of organic pollutants in a soil microbial fuel cell. Chem Eng J.

[14] Wang H, Do Park J, Ren ZJ (2015). Practical energy harvesting for microbial fuel cells: a review. Environ Sci Technol.

[15] Huang L, Chen J, Quan X, Yang F (2010). Enhancement of hexavalent chromium reduction and electricity production from a biocathode microbial fuel cell. Bioprocess Biosyst Eng.

[16] Wang X, Cai Z, Zhou Q, Zhang Z, Chen C (2012). Bioelectrochemical stimulation of petroleum hydrocarbon degradation in saline soil using U-tube microbial fuel cells. Biotechnol Bioeng.

[17] Kadivarian M, Dadkhah AA, Nasr Esfahany M (2020). Oily wastewater treatment by a continuous flow microbial fuel cell and packages of cells with serial and parallel flow connections. Bioelectrochemistry.

[18] Hamamoto K (2016). Evaluation of microbial fuel cells for electricity generation from oil-contaminated wastewater. J Biosci Bioeng.

[19] Sarmin S, Ethiraj B, Islam MA, Ideris A, Yee CS, Khan MMR (2019). Bio-electrochemical power generation in petrochemical wastewater fed microbial fuel cell. Sci Total Environ.

[20] Ieropoulos I, Winfield J, Greenman J (2010). Effects of flow-rate, inoculum and time on the internal resistance of microbial fuel cells. Bioresour Technol.

[21] Logan BE, Rossi R, Ragab A, Saikaly PE (2019). Electroactive microorganisms in bioelectrochemical systems. Nat Rev Microbiol.

[22] Doyle LE, Marsili E (2018). Weak electricigens: a new avenue for bioelectrochemical research. Bioresour Technol.

[23] Cao Y (2019). Electricigens in the anode of microbial fuel cells: pure cultures versus mixed communities. Microb Cell Fact.

[24] Bagchi S, Behera M (2021). Bioaugmentation using Pseudomonas aeruginosa with an approach of intermittent aeration for enhanced power generation in ceramic MFC. Sustain Energy Technol Assessments.

[25] Jayapriya J, Ramamurthy V (2012). Use of non-native phenazines to improve the performance of Pseudomonas aeruginosa MTCC 2474 catalysed fuel cells. Bioresour Technol.

[26] Idris MO, Kim HC, Yaqoob AA, Ibrahim MNM (2022). Exploring the effectiveness of microbial fuel cell for the degradation of organic pollutants coupled with bio-energy generation. Sustain Energy Technol Assessments.

[27] Ivanova AA, Mullaeva SA, Sazonova OI, Petrikov KV, Vetrova AA (2022). Current research on simultaneous oxidation of aliphatic and aromatic hydrocarbons by bacteria of genus Pseudomonas. Folia Microbiol (Praha).

[28] Brzeszcz J, Kaszycki P (2018). Aerobic bacteria degrading both n-alkanes and aromatic hydrocarbons: an undervalued strategy for metabolic diversity and flexibility. Biodegradation.

[29] Chamy R, Rosenkranz F (2013). Biodegradation: engineering and technology.

[30] Zarzycki-Siek J (2013). Elucidating the pseudomonas aeruginosa fatty acid degradation pathway: identification of additional fatty Acyl-CoA synthetase homologues. PLoS ONE.

[31] Jimenez-Diaz L, Caballero A, Segura A, Rojo F (2019). Pathways for the Degradation of Fatty Acids in Bacteria. Aerobic utilization of hydrocarbons, oils, and lipids.

[32] Majumder D (2014). Electricity generation and wastewater treatment of oil refinery in microbial fuel cells using Pseudomonas putida. Int J Mol Sci.

[33] Mutyala S (2021). Enabling anoxic acetate assimilation by electrode-driven respiration in the obligate aerobe, Pseudomonas putida. Bioelectrochemistry.

[34] Kaushik A, Jadhav SK (2017). Conversion of waste to electricity in a microbial fuel cell using newly identified bacteria: Pseudomonas fluorescens. Int J Environ Sci Technol.

[35] Juliano ML, Yanuaria CS, Caparanga AR, Tayo LL (2020). Low level electricity production and cod removal in wastewater using a dual chamber microbial fuel cell with pseudomonas fluorescens as biocatalyst. IOP Conf Ser Earth Environ Sci.

[36] Venkataraman A, Rosenbaum M, Arends JBA, Halitschke R, Angenent LT (2010). Quorum sensing regulates electric current generation of Pseudomonas aeruginosa PA14 in bioelectrochemical systems. Electrochem commun.

[37] Pham TH, Boon N, De Maeyer K, Höfte M, Rabaey K, Verstraete W (2008). Use of Pseudomonas species producing phenazine-based metabolites in the anodes of microbial fuel cells to improve electricity generation. Appl Microbiol Biotechnol.

[38] Rabaey K, Boon N, Höfte M, Verstraete W (2005). Microbial phenazine production enhances electron transfer in biofuel cells. Environ Sci Technol.

[39] Franco A, Elbahnasy M, Rosenbaum MA (2023). Screening of natural phenazine producers for electroactivity in bioelectrochemical systems. Microb Biotechnol.

[40] Tsipa A (2021). Iron-stimulated production and antimicrobial potential of a novel biosurfactant produced by a drilling waste-degrading pseudomonas citronellolis strain. Processes.

[41] Varnava CK (2024). Characterization, production optimization and ecotoxicity of a lipopeptide biosurfactant by Pseudomonas citronellolis using oily wastewater. Biochem Eng J.

[42] National Institute of Standards and Technology U.S. Department of Commerce https://www.nist.gov/

[43] Kamatou GPP, Viljoen AM (2017). Comparison of fatty acid methyl esters of palm and palmist oils determined by GCxGC–ToF–MS and GC–MS/FID. South African J Bot.

[44] You J, Yang S-Z, Mu B-Z (2015). Structural characterization of lipopeptides from enterobacter sp. strain N18 reveals production of surfactin homologues. Eur J Lipid Sci Technol.

[45] Sivasankara NS, Saranya P, Lokesh P, Ravindran J (2021). Identification of bioactive compounds, characterization, optimization and cytotoxic study of pyocyanin against colon cancer cell line (HT-29). J Chem Pharm Res.

[46] Gahlout M (2021). Characterization, application and statistical optimization approach for enhanced production of pyocyanin pigment by Pseudomonas aeruginosa DN9. Syst Microbiol Biomanufacturing.

[47] Koyun MT, Sirin S, Erdem SA, Aslim B (2022). Pyocyanin isolated from Pseudomonas aeruginosa: characterization, biological activity and its role in cancer and neurodegenerative diseases. Brazilian Arch Biol Technol.

[48] Hamad MNF, Marrez DA, El-Sherbieny SMR (2020). Toxicity evaluation and antimicrobial activity of purified pyocyanin from pseudomonas aeruginosa. Biointerface Res Appl Chem.

[49] Ye D (2016). Electricity production of a microbial fuel cell stack integrated into a sink drain pipe. Res Chem Intermed.

[50] Winfield J, Ieropoulos I, Greenman J, Dennis J (2011). The overshoot phenomenon as a function of internal resistance in microbial fuel cells. Bioelectrochemistry.

[51] Ieropoulos I, Gálvez A, Greenman J (2013). Effects of sulphate addition and sulphide inhibition on microbial fuel cells. Enzyme Microb Technol.

[52] Tewari BB (2000). Studies on specific surface area of different sieved fractions of nickel and cobalt ferrocyanides by dye adsorption. Rev Anal Chem.

[53] Xie J (2022). Separation and enrichment of rubidium and cesium by zinc ferrocyanide precipitation. SSRN Electron J.

[54] Zheng T (2020). Progress and prospects of bioelectrochemical systems: electron transfer and its applications in the microbial metabolism. Front Bioeng Biotechnol.

[55] Deutzmann JS, Sahin M, Spormann AM (2015). Extracellular enzymes facilitate electron uptake in biocorrosion and bioelectrosynthesis. MBio.

[56] Strycharz-Glaven SM, Snider RM, Guiseppi-Elie A, Tender LM (2011). On the electrical conductivity of microbial nanowires and biofilms. Energy Environ Sci.

[57] Behera M, Ghangrekar MM (2009). Performance of microbial fuel cell in response to change in sludge loading rate at different anodic feed pH. Bioresour Technol.

[58] Farias GA, Olmedilla A, Gallegos M-T (2019). Visualization and characterization of Pseudomonas syringae pv. tomato DC3000 pellicles. Microb Biotechnol.

[59] Nibbering NMM (2004). The McLafferty rearrangement: a personal recollection. Am Soc Mass Spectrom.

[60] Ramani K, Jain SC, Mandal AB, Sekaran G (2012). Microbial induced lipoprotein biosurfactant from slaughterhouse lipid waste and its application to the removal of metal ions from aqueous solution. Colloids Surfaces B Biointerfaces.

[61] Dauqan E, Sani HA, Abdullah A, Kasim ZM (2011). Effect of four different vegetable oils (red palm olein, palm olein, corn oil, coconut oil) on antioxidant enzymes activity of rat Liver. Pakistan J Biol Sci.

[62] Sudhakar T, Karpagam S, Shiyama S (2013). Analysis of pyocyanin compound and its antagonistic activity against phytopathogens. Int J ChemTech Res.

[63] P Priyala 2012 Pyocyanin (5-methyl-1-hydroxyphenazine) produced by Pseudomonas aeruginosa as antagonist to vibrios in aquaculture: over expression, downstream process and toxicity Cochin Univ Sci Technol [Online]. Available: https://www.researchgate.net/publication/338454130

[64] Ozdal M (2019). A new strategy for the efficient production of pyocyanin, a versatile pigment, in Pseudomonas aeruginosa OG1 via toluene addition. 3 Biotech.

[65] Wu CH, Yet-Pole I, Chiu YH, Lin CW (2014). Enhancement of power generation by toluene biodegradation in a microbial fuel cell in the presence of pyocyanin. J Taiwan Inst Chem Eng.

[66] Ben Slama H (2021). Diversity of synthetic dyes from textile industries, discharge impacts and treatment methods. Appl Sci.

[67] Natarajan S, Bajaj HC, Tayade RJ (2018). Recent advances based on the synergetic effect of adsorption for removal of dyes from waste water using photocatalytic process. J Environ Sci (China).

[68] Tara N, Siddiqui SI, Rathi G, Chaudhry SA, Inamuddin, Asiri AM (2019). Nano-engineered adsorbent for the removal of dyes from water: a review. Curr Anal Chem.

[69] Dawoud TM (2020). Characterization and antifungal activity of the yellow pigment produced by a Bacillus sp. DBS4 isolated from the lichen dirinaria agealita. Saudi J Biol Sci.

[70] El-Demerdash E (2011). Anti-inflammatory and antifibrotic effects of methyl palmitate. Toxicol Appl Pharmacol.

